# The Influence of Neurotrophic Factors BDNF and GDNF Overexpression on the Functional State of Mice and Their Adaptation to Audiogenic Seizures

**DOI:** 10.3390/brainsci12081039

**Published:** 2022-08-04

**Authors:** Angelina O. Kustova, Maria S. Gavrish, Marina A. Sergeeva, Daria A. Avlasenko, Anna O. Kiseleva, Ekaterina A. Epifanova, Alexey A. Babaev, Tatiana A. Mishchenko, Maria V. Vedunova

**Affiliations:** 1 Institute of Biology and Biomedicine, Lobachevsky State University of Nizhny Novgorod, 23 Gagarin Ave., 603022 Nizhny Novgorod, Russia; 2 Institute of Cell Biology and Neurobiology, Charité-Universitätsmedizin Berlin, Charitéplatz 1, 10117 Berlin, Germany

**Keywords:** adeno-associated virus, brain-derived neurotrophic factor, BDNF, glial cell line-derived neurotrophic factor, GDNF, mice, audiogenic seizures, neurological deficit, behavior, memory

## Abstract

The high prevalence of diagnosed cases of severe neurological disorders, a significant proportion of which are epilepsy, contributes to a high level of mortality and disability in the population. Neurotrophic factors BDNF and GNDF are considered promising agents aimed at increasing the central nervous system’s adaptive potential for the development of the epileptiform activity. Despite the pronounced neuroprotective and anticonvulsant potential, an appropriate way to stimulate these endogenous signaling molecules with minimal risk of side effects remains an open question. Herein, we assessed the safety of gene therapy using original adeno-associated viral constructs carrying the genes of neurotrophic factors BDNF and GDNF in the early postnatal period of development of experimental animals. The intraventricular injection of AAV-Syn-BDNF-eGFP and AAV-Syn-GDNF-eGFP viral constructs into newborn mice was found to provide persistent overexpression of target genes in the hippocampus and cerebral cortex in vivo for four weeks after injection. The application of viral constructs has a multidirectional effect on the weight and body length characteristics of mice in the early postnatal period; however, it ensures the animals’ resistance to the development of seizure activity under audiogenic stimulation in the late postnatal period and preserves basic behavioral reactions, emotional status, as well as the mnestic and cognitive abilities of mice after simulated stress. Our results demonstrated the safety of using the AAV-Syn-BDNF-eGFP and AAV-Syn-GDNF-eGFP viral constructs in vivo, which indicates the expediency of further testing the constructs as therapeutic anticonvulsants.

## 1. Introduction

According to the World Health Organization statistics, the number of diagnosed cases of neurological disorders with a high mortality and disability rate increases annually around the world. Epilepsy accounts for a significant share of these CNS pathologies [1]. The versatility of factors, including genetic predisposition, the negative impact of environmental factors, and a harmful lifestyle (alcoholism, smoking), makes it difficult to establish the true causes of seizure states and make a timely diagnosis and their therapeutic correction [1,2,3,4]. At present, the search for substances with neuroprotective properties in order to increase the adaptive potential of the CNS to the development of seizure activity is an urgent issue. Endogenous signaling molecules, among which brain-derived neurotrophic factor (BDNF) and glial cell line-derived neurotrophic factor (GDNF) occupy a special place, are of particular interest. These representatives of the family of small secretory proteins play a key role in the survival, differentiation, and growth of nerve cells at the stage of embryogenesis and also have a pronounced neuroprotective effect in the development of pathological conditions of the central nervous system, including epilepsy [5,6,7,8,9,10,11,12]. Neurotrophic factors BDNF and GDNF prevent neurodegeneration, promote axonal growth, maintain synaptic plasticity, and provide functions of higher nervous activity, including learning and memory [13,14,15]. A number of experimental and preclinical studies have also revealed a significant contribution of BDNF and GDNF in the activation of intrinsic mechanisms of functional recovery of damaged brain tissue after injury and other neurological disorders [16,17] which makes these neurotrophic factors an attractive therapeutic target for regenerative medicine.

Nevertheless, despite the pronounced neuroprotective and restorative potentials of neurotrophic factors BDNF and GNDF, the issue of developing strategies aimed at the effective stimulation of these endogenous signaling molecules remains unresolved. To date, no drugs with a suitable delivery method and pharmacokinetics that have the ability to act only on target molecules with a minimal risk of side effects have been developed [10,18,19].

Gene therapy based on the use of viral vectors is considered the most promising option for the clinical application of neurotrophic factors BDNF and GDNF [20]. In particular, viral vectors are actively used in the therapy of various neuronal disorders and can be directly transduced into cells secreting neurotrophic factors [21,22]. Modern vectors provide stable, long-term gene expression, have an extensive spectrum of tropism for various organs and tissues, and also exhibit relatively low pathogenicity [23]. The use of adeno-associated viruses [24,25] is considered to be the most convenient and safest option for delivering target genes to various tissues of mammals; their low immunogenicity and pathogenicity have also been proven [26]. In particular, the application of viral constructs seems to be a promising approach for offsetting the deleterious effects of a single nucleotide polymorphism of rs6265 in the BDNF gene (i.e., Val66Met polymorphism), which is considered a leading cause of deterioration in neuronal plasticity and the development of neuropsychiatric disorders [27,28]. Additionally, the expression of the BDNF gene without Val66Met polymorphism by using viral constructs provides an opportunity to normalize BDNF protein functioning, including long-term potentiation. Recent studies have revealed that overexpressing wildtype BDNF in injured Val66Met mice using an AAV-BDNF virus improved cellular, motor, and cognitive behavior outcomes, and also increased the levels of mature BDNF and phosphorylation of its key receptor TrkB [29]. However, the main problem of using adeno-associated viral vectors in clinical practice is associated with the possibility of developing an adaptive immune response and the presence of neutralizing antibodies in the patient’s body, which can be overcome by selecting a specific AAV serotype not previously observed in a certain population [30].

In this study, we evaluated the safety of using original adeno-associated viral constructs carrying the genes of neurotrophic factors BDNF and GDNF in the early postnatal development of experimental animals. We performed intraventricular injection of AAV-Syn-BDNF-eGFP and AAV-Syn-GDNF-eGFP viral constructs into newborn mice and assessed the weight and body length characteristics of individuals during the first three weeks of postnatal development. Next, we analyzed the risks of developing seizure activity in mice when provoking audiogenic seizures, followed by a series of behavioral tests to analyze general motor and orienting-exploratory activities, as well as mnestic and cognitive functions. Expression features of neurotrophic factors BDNF and GDNF and their key receptors (TrkB, GFRα1) in the brain tissues of mice in late postnatal development were studied using the real-time PCR.

## 2. Materials and Methods

### 2.1. Research Object

Hybrid mice of two lines C3H and C57Bl6 (C3H+C57Bl6), obtained by the crossing scheme described in our previous work [31], were the objects of study. The mice were housed in a certified SPF vivarium at Lobachevsky University. All experimental procedures were approved by the Bioethics Committee of Lobachevsky University and carried out in accordance with Act 708n (23 082010) of the Russian Federation National Ministry of Public Health, which states the rules of laboratory practice for the care and use of laboratory animals, and the Council Directive 2010/63 EU of the European Parliament (22 September 2010) on the protection of animals used for scientific purposes.

### 2.2. Scheme of the Experiment

**Figure 1:**
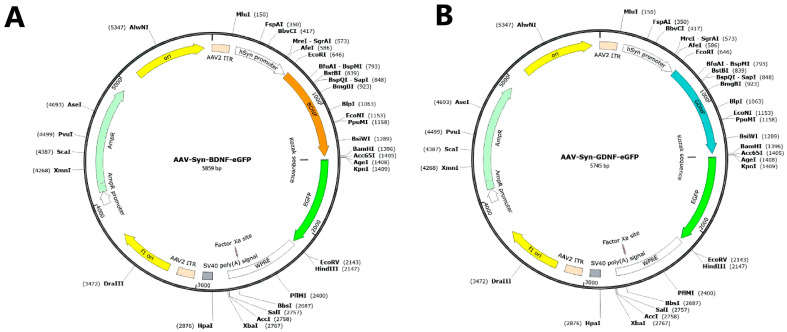
Maps of pAAV-Syn-BDNF-eGFP (**A**) and pAAV-Syn-GDNF-eGFP (**B**) plasmid vectors (constructed using the SnapGene Viewer 3.1.2 software (from Insightful Science; available at snapgene.com) (GSL Biotech LLC, San Diego, CA, USA).

Newborn mice at the P0 stage were intraventricularly injected with the original adeno-associated viral vectors AAV-Syn-BDNF-eGFP [32] and AAV-Syn-GDNF-eGFP, carrying gene sequences of neurotrophic factors and green fluorescent protein eGFP (Figure 1).

Injection with viral vectors was carried out according to the protocol described by Kim et al. [33,34]. The timing of injection (the first day after birth) is based on the fact that the ventricular ependymal lining, not yet fully formed, does not prevent neuronal transduction throughout the brain [33,34]. Experimental animals were divided into the following groups: sham (no injection), mice that were not injected; control (PBS) mice that were injected with phosphate-buffered saline (PBS); and two experimental groups of animals with a viral injection of AAV-Syn-BDNF-eGFP and AAV-Syn-GDNF-eGFP vectors, respectively.

During the following periods of postnatal development, weight and body length characteristics were measured in the animals.

On day 21 of the postnatal period, audiogenic seizures were provoked in mice. Subsequently, the neurological status, motor, and orienting-exploratory activity were assessed using the Open Field test, and mnestic and cognitive functions were analyzed using the conditioned passive avoidance reflex (CPAR) test. The expression levels of neurotrophic factors BDNF and GDNF and their key receptors (TrkB, GFRα1) in brain tissues were determined by real-time PCR on day 30 of the postnatal period.

### 2.3. Audiogenic Seizures Model

On day 21 of the postnatal period, audiogenic seizures were provoked in mice in accordance with the study of Semiokhina et al. [35]. Each animal was placed in the soundproof box, and after a 1min adaptation, a single electromechanical bell with a sound intensity of 110 dB was given. The sound signal was turned off immediately after the onset of a seizure or 1 min after the bell was turned on.

The intensity of seizure activity manifestation in response to sound stimulation was assessed according to the Krushinsky scale [31,36]. In addition to assessing the intensity of the seizures, the number of fatalities was also assessed.

### 2.4. Neurological Status Assessment

The analysis of the development of neurological deficits in animals was carried out according to the Scale for the Assessment of Neurological Deficits in Small Laboratory Animals, with modifications [37,38]. In each animal, ten involuntary innate behavioral responses were recorded, each of which was assessed by a scoring system. The scale includes ten tests to identify features of motor activity, trajectory and coordination of movements, the severity of reflexes, muscle tone, and the presence/absence of ptosis and exophthalmos. If the animal performed the test, the score was 0 points; performed partially—1 point; if no reaction was observed—2 points. Based on the test results, the scores were summarized and interpreted according to the following gradation: 10–20 points indicating severe CNS injury; 6–9 points—moderate CNS damage; and 1–5 points—slight CNS damage.

### 2.5. Open Field Test

The study of the general motor and orienting-exploratory activity of the animals was carried out using the OpenField equipment (IR Actimeter) (Panlab, Barcelona, Spain) and PanLabActiTrack (Panlab, Barcelona, Spain) and Stoelting (Stoelting Co., Whood Dale, IL, USA) software. The Panlab Infrared (IR) Actimeter includes a 2D square frame and an infrared beam system to detect animal movements. The following main behavioral responses were analyzed: the total distance covered, the distance covered in the center and on the periphery of the arena, the number of upright postures, the number of acts of defecation, urination, and time spent in the center of the arena, which characterizes the emotional state of the animal.

### 2.6. Test of Conditioned Passive Avoidance Reflex (CPAR)

A chamber (60 cm × 20 cm × 25 cm) with an electrified slatted floor, divided by a partition into darkened and lighted sections (Shuttle Box LE918; Panlab Harvard Apparatus, Barcelona, Spain), was used to study the ability of animals to learn [31]. The animal was placed in the brightly lit section, and the latent period of transition to the dark compartment was measured. After the animal entered the dark section of the chamber, an electrical impulse (0.08 mA) was applied for 5 s as a stimulus. Twenty-four hours later, a second test was performed to assess the time of transition to the dark section. The duration of the first training and repeated testing was 180 s.

### 2.7. Real-Time PCR

The level of expression of neurotrophic factors (BDNF, GDNF) and their key receptors (TrkB, GFRα1) in the brain tissues of animals was assessed by real-time PCR analysis.

Total RNA from the cerebral cortex and hippocampus of the mouse brain was isolated by phenol–chloroform extraction using ExtractRNA (Eurogen, Russia) according to the manufacturer’s protocol. MMLV RT kit and random primer (Eurogen, Russia) were used for reverse transcription.

Real-time PCR was performed using a ready-made reaction mixture qPCRmix-HS SYBR+LowROX (Eurogen, Russia).

The following sequences of primer pairs were used:

Oaz1_fw 5′-AAGGACAGTTTTGCAGCTCTCC-3′;

Oaz1_rv 5′-TCTGTCCTCACGGTTCTTGGG-3′;

BDNF_fw 5′-CCCAACGAAGAAAACCATAAGGA-3′;

BDNF_rv 5′-CCAGCAGAAAGAGTAGAGGAGGCT-3′;

GDNF_fw 5′-CCTTCGCGCTGACCAGTGACT-3′;

GDNF_rv 5′-GCCGCTTGTTTATCTGGTGACC-3′;

TrkB_fw 5′-TTTCCGCCACCTTGACTTGTCT-3′;

TrkB_rv 5′-GTCGGGGCTGGATTTAGTCTCC-3′;

GFRα1_fw 5′-TGTCTTTCTGATAATGATTACGGA-3′;

GFRα1_rv 5′-CTACGATGTTTCTGCCAATGATA-3′.

qPCR conditions: 50 °C for 2 min, 95 °C for 10 min, 40 cycles of 95 °C for 15 s, and 60 °C for 60 s on an Applied Biosystems 7500 thermocycler (Applied Biosystems, Thermo Fisher Scientific, Waltham, MA, USA).

The results were processed by the ΔΔCt method using samples obtained from the control (not injected) group of animals, in which the expression level was taken as one. Oaz1 was used as a reference gene.

### 2.8. Statistical Analysis

Statistical analysis was performed using GraphPad Prism v.9.3.1.471 (San Diego, CA, USA). The results are presented as the mean ± standard error of the mean (SEM). The Shapiro–Wilk test was used for normal distribution analysis. Differences between groups were considered significant if the corresponding *p*-value was less than 0.05.

## 3. Results

**Figure 2:**
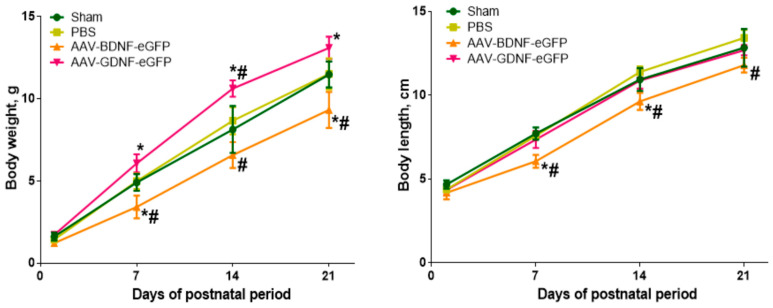
Body mass and body length values of newborn mice during three weeks of the postnatal period. *-vs. “Sham”, #-vs. “PBS”, *p*< 0.05, one-way ANOVA with multiple comparison test.

First, we evaluated the effects of the use of the AAV-Syn-BDNF-eGFP and AAV-Syn-GDNF-eGFP viral constructs on the development of animals in the postnatal period. An assessment of the weight and body length characteristics showed that the newborn mice of the control groups gradually gained body length and weight during the first three weeks of the postnatal period (Figure 2). Weekly weight gain in the “Sham” and “PBS” groups averaged 3.3 g, and the increase in body length averaged 2.7 cm for “Sham” and 3 cm for the “PBS” group, respectively. There were no significant differences in the weight and body length characteristics of the control groups.

Newborn mice of the “AAV-BDNF-eGFP” group showed a slight developmental delay. Animals of this experimental group gained body length and weight less intensively. The weekly increase in body weight averaged 2.8 g, and the increase in body length averaged 2.5 cm. A significant decrease in the values of weight and body length parameters relative to the control groups was found starting from day 7 of the postnatal period and over the next two weeks.

On the contrary, newborn mice of the “AAV-GDNF-eGFP” group gained weight rapidly. A significant increase in body weight relative to the control values was observed starting from day 7 of the postnatal period and over the next two weeks. Weekly weight gain in the “AAV-GDNF-eGFP” group averaged 3.8 g. There were no significant differences in body length parameters relative to the control groups.

On day 21 of the postnatal period, audiogenic seizures were provoked in mice. The studies showed that the audiogenic stimulation of hybrid individuals, both injected and not injected with viral constructs, did not cause the development of seizure activity. In the animals of control and experimental groups, the intensity of seizure activity in response to sound stimulation was 0 points (no reaction to sound for 1 min according to the Krushinsky scale) in 100% of the cases.

The provocation of audiogenic seizures did not lead to the development of neurological deficit in animals. On the day following audiogenic stimulation, in the “AAV-BDNF-eGFP” and “AAV-GDNF-eGFP” groups, the neurological deficit was 1.1 ± 0.2 and 1.7 ± 0.3 points, respectively, which did not significantly differ from the values of the sham 2.3 ± 0.3 and control “PBS” group 2.3 ± 0.3 (*p* > 0.05, the Kruskal–Wallis test).

The analysis of behavioral responses in the Open Field test did not reveal significant changes in the general motor and orienting-exploratory activity of the animals in the control group and groups using viral constructs (Table 1).

After provoking audiogenic seizures, we also assessed the learning ability and cognitive functions of the mice by using the passive avoidance test (Table 2). The studies revealed that the animals of the experimental groups retained the ability to learn.

During the training session, the mice entered the dark chamber section and tended to leave the brightly lit space, following the mink reflex. There were no significant differences with the “Sham” group. The latent period of movement to the dark section in retesting significantly increased in all experimental groups compared to the values of the training session. No significant differences relative to the “Sham” group were shown. These data demonstrate that the mice preserved a memorial trace in response to electrical stimulus.

**Figure 3:**
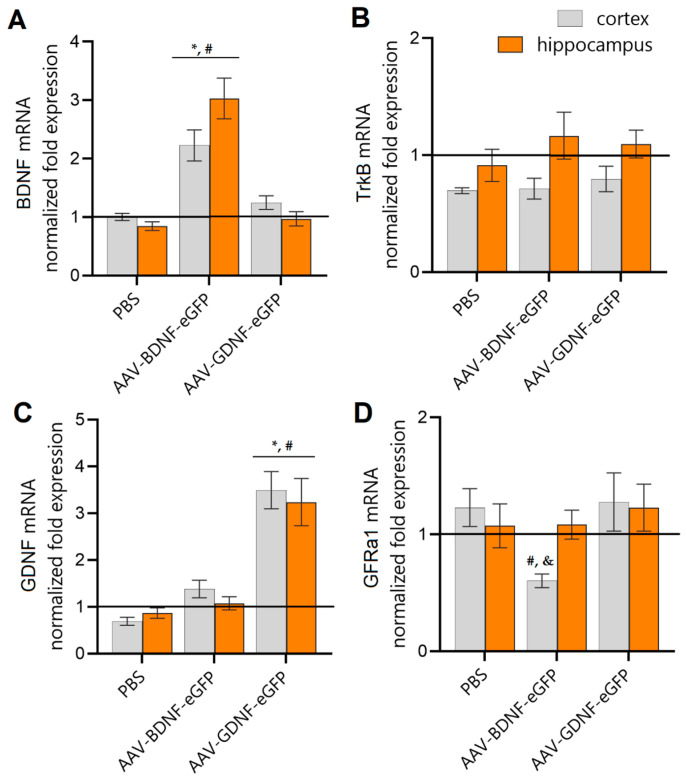
Assessment of the level of mRNA expression of neurotrophic factors BDNF (**A**) and GDNF (**C**), and their key receptors TrkB (**B**) and GFRa1 (**D**), in the cerebral cortex and hippocampus of mice 7 days after the provocation of audiogenic seizures. Data were normalized relative to values of the “Sham” group. *-vs. “Sham”, #-vs. “PBS”, &-vs. levels in the hippocampus within the group, *p* < 0.05, Kruskal-Wallis test with Dunn’s multiple comparisons test.

One week after provoking audiogenic seizures, the expression of the genes of neurotrophic factors BDNF and GDNF, and their key receptors (TrkB, GFRα1), in the brain tissues of the hybrid line of mice was evaluated (Figure 3). When analyzing the expression of the BDNF, GDNF, TrkB, and GFRα1 genes, threshold values were set relative to the mice of the sham group.

Previous immunohistochemical analysis revealed the confident expression of eGFP protein in the mouse brain tissue starting from the third week after transduction with the AAV-Syn-BDNF-eGFP and AAV-Syn-GDNF-eGFP viral constructs [39]. Herein, we showed that the use of the AAV-Syn-BDNF-eGFP and AAV-Syn-GDNF-eGFP viral constructs provided stable overexpression of the target genes. Thus, endogenous stimulation of the neurotrophic factor BDNF by using the adeno-associated virus AAV-Syn-BDNF-eGFP provides increased mRNA levels in the hippocampus and cerebral cortex of mice by an average of 2.2-fold and 3-fold, respectively, compared to the sham group (Figure 3). Endogenous stimulation of BDNF had no significant effect on the expression level of the neurotrophic factor GDNF. It is interesting to note that in the “AAV-BDNF-eGFP” group, the level of expression of TrkB, the key receptor for the neurotrophic factor BDNF, in the cerebral cortex did not significantly differ from the values of the sham group, while the level of expression of GFRα1, the key receptor for the neurotrophic factor GDNF, was reduced by 0.6-fold.

Endogenous stimulation of the neurotrophic factor GDNF by application of adeno-associated virus AAV-Syn-GDNF-eGFP provides an increased expression of the gene of interest in the cerebral cortex and hippocampus by an average of 3.4-fold and 3.2-fold relative to the sham group. However, in contrast to the action of the AAV-Syn-BDNF-eGFP viral vector, the use of AAV-Syn-GDNF-eGFP does not affect the expression of either the key receptor (GFRα1) or the neurotrophic factor BDNF and its TrkB receptor.

## 4. Discussion

Currently, there is an annual increase in the number of diagnosed cases of seizure states that have many phenotypic manifestations and are the ultimate common conductor of numerous pathophysiological processes in the CNS [1,2,3,40,41]. The development of seizure activity leads to significant changes at the cellular or synaptic level, which result in the formation of abnormal neural networks with increased excitability. The consequences of a change in the balance between excitatory and inhibitory signals, as well as the operation of voltage-dependent ion channels, are neuronal death, active gliosis, increased permeability of the blood–brain barrier, the development of inflammation, and neurodegenerative processes [2,40,42,43]. The occurrence of seizures is unpredictable, which increases the risk of injury, disability, and mortality, and negatively affects the patient’s mental health, often leading to anxiety, depression, and cognitive impairment [40,44,45]. In this regard, there is an urgent need to develop methods for early diagnosis and effective methods for the treatment of seizure states.

The use of neurotrophic factors BDNF and GDNF is considered one of the promising therapeutic strategies aimed at increasing the adaptive potential of the CNS for the development of seizure activity. However, the issue of developing an optimal method for stimulating neurotrophic factors and the safety of their use as therapeutic agents at the organismic level remains open. Thus, a number of studies have shown that an increase in the concentration of neurotrophic factors can provoke the development of seizure activity and disrupt the development of the vertebrate nervous system [22]. It was found that the neurotrophic factor BDNF has an excitatory effect on neuron cultures and brain slices of animals. A single application of BDNF to the brain can provoke the development of seizure activity in mice, while a chronic infusion of BDNF can lead to a decrease in neuronal excitability [46]. A similar contradiction is observed in the case of the neurotrophic factor GDNF [47]. Increased expression of GDNF in the hippocampus can induce seizures [19]. On the other hand, a slight increase in GDNF expression can have a pronounced antiepileptic effect [12].

In the present study, we evaluated the safety of overexpression of neurotrophic factors BDNF and GDNF by using adeno-associated viral constructs carrying the genes of neurotrophic factors BDNF and GDNF in the early postnatal development of a hybrid mouse line. We have shown that intraventricular injection of AAV-Syn-BDNF-eGFP and AAV-Syn-GDNF-eGFP provides persistent overexpression of target genes in the hippocampus and cerebral cortex of mice during the four weeks of postnatal development. At the same time, overexpression of the neurotrophic factor GDNF through the use of the AAV-Syn-GDNF-eGFP viral construct does not significantly affect the expression level of the neurotrophic factor BDNF, and TrkB and GFRα1, the key receptors of these neurotrophic factors. Against the background of BDNF neurotrophic factor overexpression achieved by using the viral construct AAV-Syn-BDNF-eGFP, a decrease in the expression level of GFRα1, the neurotrophic factor GDNF receptor, in the cerebral cortex was found. Taking into account the possibility of a putative antagonistic action of neurotrophic factors BDNF and GDNF under stress [48], a decrease in the level of GDNF/GFRα1 signaling is apparently associated with the activation of compensatory mechanisms aimed at maintaining a balance between neurotrophic factors, preventing the development of pathological reactions. We have shown that the use of viral constructs affects the weight and body length characteristics of mice. When AAV-Syn-BDNF-eGFP was applied, there was a reduced rate of weight gain and growth rate, while individuals in the “AAV-Syn-BDNF-eGFP” group gained weight rapidly starting from the second week of the postnatal period. Despite changes in weight and body length characteristics, mice were resistant to stress. Audiogenic stimulation did not provoke the development of seizure activity in animals. No neurological deficit was observed in the mice, as well as no pronounced changes in basic behavioral reactions, emotional status, and mnestic and cognitive abilities after the simulated stress. Our detailed description of the phenotypic status of the hybrid line allowed us to find that the use of the AAV-Syn-BDNF-eGFP and AAV-Syn-GDNF-eGFP viral constructs ensures the stable overexpression of target genes in brain tissues and is safe for use at the organismic level. Further studies will focus on the effectiveness of AAV-Syn-BDNF-eGFP and AAV-Syn-GDNF-eGFP as therapeutic agents in provoking seizure activity in mutant mice with a genetic predisposition to epilepsy.

## 5. Conclusions

The use of the AAV-Syn-BDNF-eGFP and AAV-Syn-GDNF-eGFP viral constructs provides persistent overexpression of target genes in the hippocampus and cerebral cortex in vivo for four weeks after intraventricular injection. The application of viral constructs has a multidirectional effect on the weight and body length characteristics of mice in the early postnatal period; however, it ensures the animals’ resistance to the development of seizure activity during audiogenic stimulation in the late stages of postnatal development while maintaining basic behavioral reactions, emotional status, and mnestic and cognitive abilities of mice after simulated stress. The data obtained indicate that it is safe to use the AAV-Syn-BDNF-eGFP and AAV-Syn-GDNF-eGFP viral constructs in vivo, which justifies the expediency of further testing the constructs as therapeutic anticonvulsants.

## Figures and Tables

**Table 1:** Parameters of behavioral reactions of mice in the Open Field test after the provocation of audiogenic seizures.

*A: Parameters of locomotor activity*					
**Mouse Group**	**Distance Traveled in the Arena, cm**			**Time in the Arena Center, s**	**Number of Upright Postures**
	**Periphery**	**Center**	**Total**		
Sham	841.2 ± 247.4	105.2 ± 28.7	973.3 ± 161.3	67.7 ± 34.8	29.8 ± 5.7
PBS	1526.2 ± 172.5	120.1 ± 11.5	1421.5 ± 162.8	22.9 ± 12.9	29.4 ± 4.5
AAV-BDNF-eGFP	1005.5 ± 174.0	130.7 ± 43.5	1001.26 ± 160.8	32.7 ± 16.5	20.4 ± 5.4
AAV-GDNF-eGFP	762.1 ± 242.8	92.7 ± 30.6	817.6 ± 158.1	44.4 ± 31.1	16.7 ± 6.2
*B: Emotional status characteristics*					
**Mouse Group**	**Acts of Urination**			**Acts of Defecation**	
Sham	1.6 ± 0.4			2.7 ± 0.6	
PBS	2.6 ± 0.5			2.7 ± 0.8	
AAV-BDNF-eGFP	2.1 ± 0.5			2.7 ± 0.4	
AAV-GDNF-eGFP	1.7 ± 0.5			3.3 ± 0.9	

*p* > 0.05, the Kruskal–Wallis test.

**Table 2:** Efficiency of reproduction of the conditioned passive avoidance reflex in mice after the modeling of audiogenic seizures.

Mouse Group	Latent Period of Movement to the Dark Chamber Section, s	
	Training Session	Retesting
Sham	40.9 ± 11.8	155.7 ± 9.3
PBS	32.1 ± 6.2	162.9 ± 9.1
AAV-BDNF-eGFP	45.1 ± 9.9	167.6 ± 9.6
AAV-GDNF-eGFP	50.9 ± 6.7	179.7 ± 0.3

*p* > 0.05, the Kruskal–Wallis test.

## Data Availability

The data used to support the findings of this study are available from the corresponding author upon request.
